# Acute care surgery: a means for providing cost-effective, quality care for gallstone pancreatitis

**DOI:** 10.1186/s13017-017-0128-3

**Published:** 2017-04-28

**Authors:** Patrick B. Murphy, Dave Paskar, Richard Hilsden, Jennifer Koichopolos, Tina S. Mele, on behalf of Western Ontario Research Collaborative on Acute Care Surgery

**Affiliations:** 1 Division of General Surgery, Department of Surgery, Schulich School of Medicine and Dentistry, Western University, London, Canada; 2 Divisions of General Surgery Trauma & Critical Care Medicine, University of Toronto, Toronto, Canada; 3 Divisions of Critical Care, Department of Surgery, Schulich School of Medicine and Dentistry, Western University, London, Canada

**Keywords:** Acute care surgery, Quality, Cost effectiveness, Gallstone pancreatitis, Cholecystectomy

## Abstract

**Background:**

Modern practice guidelines recommend index cholecystectomy (IC) for patients admitted with gallstone pancreatitis (GSP). However, this benchmark has been difficult to widely achieve. Previous work has demonstrated that dedicated acute care surgery (ACS) services can facilitate IC. However, the associated financial costs and economic effectiveness of this intervention are unknown and represent potential barriers to ACS adoption. We investigated the impact of an ACS service at two hospitals before and after implementation on cost effectiveness, patient quality-adjusted life years (QALY) and impact on rates of IC.

**Methods:**

All patients admitted with non-severe GSP to two tertiary care teaching hospitals from January 2008–May 2015 were reviewed. The diagnosis of GSP was confirmed upon review of clinical, biochemical and radiographic criteria. Patients were divided into three time periods based on the presence of ACS (none, at one hospital, at both hospitals). Data were collected regarding demographics, cholecystectomy timing, resource utilization, and associated costs. QALY analyses were performed and incremental cost effectiveness ratios were calculated comparing pre-ACS to post-ACS periods.

**Results:**

In 435 patients admitted for GSP, IC increased from 16 to 76% after implementing an ACS service at both hospitals. There was a significant reduction in admissions and emergency room visits for GSP after introduction of ACS services (*p* < 0.001). There was no difference in length of stay or conversion to an open operation. The implementation of the ACS service was associated with a decrease in cost of $1162 per patient undergoing cholecystectomy, representing a 12.6% savings.

The time period with both hospitals having established ACS services resulted in a highly favorable cost to quality-adjusted life year ratio (QALY gained and financial costs decreased).

**Conclusions:**

ACS services facilitate cost-effective management of GSP. The result is improved and timelier patient care with decreased healthcare costs. Hospitals without a dedicated ACS service should strongly consider adopting this model of care.

## Background

Dedicated ACS services continue to be implemented in North America and around the globe. Increasingly comparable to trauma systems, ACS is a growing field with developing fellowships [1], centralization of care, and on-going research particularly with respect to appendicitis [2] and acute biliary disease [3, 4]. The benefits of ACS services are many, and include the optimization of health care delivery from the perspectives of surgeons, patients, and hospitals; these positive changes have been summarized and reported in two systematic reviews to date [5, 6].

GSP is one of the most common gallstone-related emergency general surgery (EGS) conditions, the definitive management of which is index cholecystectomy (IC) [7–13]. IC is recommended as the burden of recurrence requiring additional emergency room visits and re-admissions to hospital is high if definitive management (cholecystectomy) is not provided on the index admission [14–21]. A recent randomized trial, the PONCHO trial, has confirmed the safety and efficacy of index cholecystectomy for non-severe gallstone pancreatitis [22]. A Cochrane meta-analysis further suggests that early IC (<72h) for GSP is safe [23]. However, the facilitation of IC remains a challenge in many centers [15–19]. Lack of emergency general surgical resources, specifically dedicated operating room (OR) time, is the most significant barrier to the provision of early definitive care (IC) [24]. Additional challenges include surgeon acceptance of index (vs. delayed) cholecystectomy and competition with elective practice demands. As such, significant variation persists with respect to the management of acute biliary disease, gallstone pancreatitis in particular [25].

Our previous work has demonstrated an ACS service that can facilitate IC for GSP, leading to decreased GSP recurrence and subsequently reduced emergency room (ER) visits and re-admissions [4]. The cost of ACS provision has been described for acute cholecystitis, and in general, results in cost savings [24–26]. However, no study, to our knowledge, has reported on the impact of patient quality of life, nor performed a formal health economic analysis, following the implementation of an ACS service.

It is well established that delay of definitive management confers an approximately 25% risk of re-admission for gallstone-related illness prior to elective cholecystectomy [14–23]. This potentially impacts patient quality of life with respect to pain, anxiety, and lost days of work and other forms of social functioning. A model-based cost-utility analysis using data from the recent Cochrane review has suggested IC, particularly within 72 h, is cost effective for mild acute gallstone pancreatitis [26].

In our first study, a notable increase in IC rate for GSP at a single center was noted following the initiation of an ACS service (with a non-ACS center serving as a time-control). This second institution has since added an ACS service, allowing us to examine whether the improved outcomes for GSP can be replicated. In addition, we sought to assess the sustainability of a high IC rate within a mature ACS service over time, by continuing to track performance at the first site. Moreover, we investigated the fiscal and quality of life impacts associated with implementing an ACS service by performing a formal health economic analysis from the perspective of the GSP population. We hypothesize a sustainable rate of IC in keeping with our previous results after implementing ACS as well as cost savings and improved quality of life for patients compared to non-index cholecystectomy.

## Methods

Ethics approval was obtained from the Health Sciences Research Ethics Board at Western University (HSREB# 104525). The study was conducted at London Health Sciences Centre, a tertiary care, academic institution comprised of two hospital campuses (site A and site B for the purposes of this study) in London, Ontario, Canada. In July 2010, one campus (site A) implemented an ACS service and in July 2014, site B implemented a similar service. A complete description of our ACS model is available [4]. Briefly, in our model, a surgeon suspends his or her elective practice to cover emergency surgical consults, admissions, and surgeries, both through the emergency department and via other in-hospital services, for 1 week. Site A has dedicated daily emergency operative time while site B shares time with other surgical services.

We retrospectively reviewed the charts of all patients admitted to general surgery at both hospitals for the period of January 2008 to May 2015 with an initial diagnosis of pancreatitis. This group of patients was then further refined to include only those with GSP. Our inclusion criteria were as follows: age 18 years old or older, a lipase greater than 300 U/L, had not undergone ERCP within 7 days prior to presentation, and the presence of gallstones on imaging. In order to identify all potential candidates for IC, patients were excluded only if they were pregnant and/or had an intensive care unit (ICU) admission within 48 h of admission to a hospital. ICU admission was used to exclude patients with probable severe pancreatitis and/or had significant co-morbidities, and thus likely not suitable candidates for IC. All patient health records were complete for all parameters of interest, and thus, no patient had missing data. In addition, our network of partner community hospitals shares an electronic health record with our institution, so we were able to detect if patients sought surgical care at these sites.

We divided this population into three time periods. Period 1 (January 1, 2008, to June 30, 2010; 2.5 years) represents when neither hospital had an ACS service. During period 2 (July 1, 2010, to June 30, 2014; 4 years), an ACS service was only present at site A. Finally, period 3 (July 1, 2014, to May 15, 2015; 10.5 months) represents the era when both sites had an ACS service.

Descriptive statistics including demographics, use of ERCP (index or not), time to OR from ER presentation, OR duration, conversion to open cholecystectomy, and site of index admission were collected. Re-admission and repeat ER visits rates for GSP, gallstone-related complaints or postoperative concerns were also tracked.

### Cost analysis

The cost effectiveness was evaluated from the perspective of a single government payer, which is consistent with the public healthcare systems employed in Canada. The time horizon for the cost effectiveness analysis was either 1 year or the actual duration of illness related to gallstone pancreatitis, for patients whose disease experience in the study extended beyond 1 year. No patients in this study were awaiting management beyond 2 years. A discount rate of 3.5% per year was applied to both health effects and costs, in line with the National Institute for Health and Care Excellence (NICE) guidelines for health economic analysis [27]. Patients who presented with GSP but did not ultimately receive surgical management were excluded from the cost analysis. These patients were excluded as it cannot be assumed that non-surgical management restored these patients to their pre-morbid health state.

Costs for providing management to each individual patient were estimated using financial data available from the Ontario Case Costing Program which contains publicly available data, maintained by the Province of Ontario, Table 1 [28]. This database contains both direct and indirect costs of managing patients with particular diagnoses and of performing certain procedures, as reported by participating hospitals. Using the database, the cost of each component of care for a patient with GSP was estimated, and then was applied to each individual patient in this study, Table 1.

**Table 1:** Cost and QALY data

Cost	Value	Source
Outpatient laparoscopic cholecystectomy	$1389	Avg direct cost (OCCP)
Inpatient open cholecystectomy	$10,423	Avg direct cost (OCCP)
Inpatient laparoscopic cholecystectomy	$4349	Avg direct cost (OCCP)
ERCP	$839	Avg direct cost (OCCP)
Ultrasound abdomen	$356	Avg direct cost (OCCP)
CT abdomen	$491	Avg direct cost (OCCP)
ER visit acute cholecystitis	$286	Avg direct cost (OCCP)
Hospital admission cost per day^a^	$776	Derived from OCCP data
Surgeon fee	$478	2011 OMA SOB
Anesthesia unit fee	$15.01	2011 OMA SOB
Cholecystectomy units	7 + time units	2011 OMA SOB
QALY—health states	Value	Source
No biliary disease	1	
Laparoscopic cholecystectomy	0.91	Bass, et. al. 1993 [30]
Recurrent biliary colic	0.8	Cook, et. al. 1993 [29]
Open cholecystectomy	0.77	Bass, et. al. 1993 [30]
Acute pancreatitis	0.44	Cook, et. al. 1993 [29]
Death	0	

*OCCP* Ontario Case Costing Program 2011 values, *OMA SOB* Ontario Medical Association Schedule of Benefits

^a^Includes indirect costs related to hospital admission

### Quality analysis

Quality-adjusted life years (QALY) were used to evaluate health effects. All patients were assigned a QALY of 1 (full health) to represent their health condition prior to developing gallstone pancreatitis. The health multipliers for health states associated with GSP were as follows: no biliary disease—1.0, laparoscopic cholecystectomy—0.91 [29], recurrent biliary colic—0.80 [29], open cholecystectomy—0.77 [30], and acute pancreatitis—0.44 [29]. While admitted to hospital, patients were assigned the acute pancreatitis multiplier. Patients that were discharged from hospital, but who did not have IC, were assigned the recurrent biliary colic QALY multiplier to represent failure to return to normal health. Finally, patients treated surgically were assigned the QALY multiplier that represents the surgery they received for the remaining study time horizon. An incremental cost effectiveness ratio (ICER) comparing the study time periods was calculated. As per health economic analysis convention, it was predetermined that only positive ICERs would be reported in the final analysis. Outcomes that both save money and improve clinical outcomes are “dominant strategies” whose magnitude of effect cannot be described by the ICER, nor are the “dominated strategies” that result in greater economic expenditures and lead to worse outcomes. Both of these situations result in negative ICERs whose magnitude offers no practical relevance in terms of decision-aiding.

### Statistical analysis

Continuous parameters were expressed as mean with standard deviation and nonparametric data were expressed as median and interquartile range. Categorical data were expressed as proportions. We performed one-way ANOVA analysis of variance, Pearson Chi-squared or Mann-Whitney *U* tests to determine statistical significance, with an alpha of 0.05, depending on the nature of the variable. Multivariate logistic regression was used to determine factors influencing IC performance. The primary clinical outcome was IC rate, and the primary cost end-point was an incremental cost effectiveness ratio (ICER). The student’s *t* test was used to compare each time period (periods 2 and 3) to the reference period of neither site having ACS (period 1). Secondary end-points included length of stay, OR duration, number of ER visits, and re-admissions for gallstone pancreatitis. SPSS Version 20 (IBM Inc., 2011) was used as a statistical platform.

## Results

**Fig. 1:**
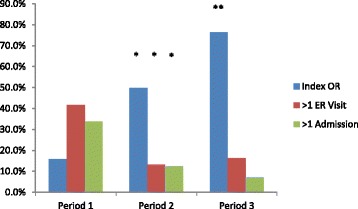
Rate of index cholecystectomy, emergency room visits, and admissions across the three time periods. (*<0.05 compared to period 1, **<0.05 compared to period 2

There were a total of 435 patients admitted to general surgery with GSP during the study periods, Table 2. There was no difference with respect to the sex and age of patients in any of the periods. The implementation of an ACS service drastically increased the rate of IC from 16% in period 1 to 76% in period 3 when both sites had an ACS service; Table 3, Fig. 1. When examining the sites separately, there was an increase in IC rate from 19 to 70% (*p* < 0.001) and 25 to 83% (*p* < 0.001) following ACS implementation at sites A and B, respectively. The median ER to OR time for IC was 3.3 days (2.4–4.6) and for elective operation was 53.6 days (18.6–101.4) (*p* < 0.001). In a multivariate model of predicting IC that included age, sex, site of admission, and presence of an ACS service, only age (OR 0.97; 95% CI 0.95–0.98) and the presence of an ACS service (OR 13.6; 95% CI 7.5–24.8) were statistically significant.

**Table 2:** Patient demographics of the three periods, based on the presence of an ACS service

	Period 1	Period 2	Period 3	*p* value
Number, n	139	241	55	–
Site A, *n* (%)	73 (53)	127 (53)	32 (58)	0.74
Age, year, mean (SD)	55 (21)	58 (20)	57 (21)	0.45
Male, *n* (%)	50 (36)	97 (40)	23 (40)	0.65

Period 1: 2008–2010 (no ACS at either site)

Period 2: 2010–2014 (ACS at site A)

Period 3: 2014–2015 (ACS at both sites)

**Table 3:** Comparison of clinical outcomes and total cost between three time periods, based on the presence of an ACS service

	Period 1	Period 2	Period 3	*p* value
Number, *n*	139	241	55	–
Index OR, *n* (%)	22 (16)	120 (50)	42 (76)	<0.001
Elective OR, *n* (%)	85 (61)	67 (28)	6 (11)	<0.001
Inpatient ERCP, *n* (%)	61 (44)	87 (36)	16 (29)	0.11
ER to OR, d, mean (SD)	95 (194)	32 (66)	23 (107)	<0.001
OR duration, minutes	65 (32)	68 (29)	63 (34)	0.44
Open, *n* (%)	6 (4)	9 (4)	0 (0)	–
Postoperative stay, d, mean (SD)	1.5 (1.8)	1.4 (5.7)	1.1 (1.9)	0.87
Total LOS on index admission, days, mean (SD)	5 (3)	6 (9)	5 (3)	0.10
More than one admission for GSP, *n* (%)	47 (34)	30 (12)	4 (7)	<0.001
More than one ER visit for GSP, *n* (%)	58 (42)	32 (13)	9 (16)	<0.001
Time of index OR				
Mon–Fri (7 a.m.–5 p.m.)	10 (46)	73 (61)	26 (62)	0.675
Mon–Fri (5 p.m.–Midnight)	2 (9)	9 (8)	2 (5)	
Mon–Fri (Midnight–7 a.m.)	0 (0)	0 (0)	0 (0)	
Weekend	10 (46)	38 (32)	14 (33)	
Total cost (2011 dollars), mean (SD)	$9255 (324)	$9307 (648)	$8093 (505)	<0.001

Period 1: 2008–2010 (no ACS at either site)

Period 2: 2010–2014 (ACS at site A)

Period 3: 2014–2015 (ACS at both sites)

*LOS* Length of stay, *ERCP* endoscopic retrograde cholangiopancreatography, *ER* emergency room, *OR* operating room

**Fig. 2:**
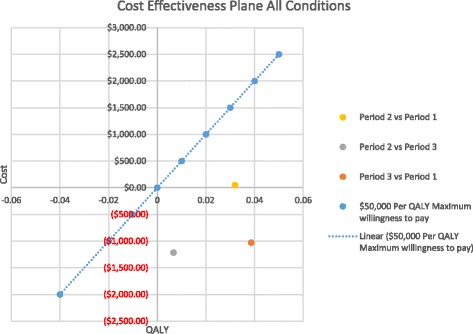
Cost effectiveness plane for each time period; period 3 (ACS at both sites favored)

A total of 342 patients received definitive surgical management for GSP. The re-admission rate and the number of ER visits for GSP were significantly reduced with the addition of an ACS service, Table 3 and Fig. 1. When considering index, elective, and non-surgery groups, the rates of re-admission were 3, 30, and 29%, respectively (*p* < 0.001). OR duration, postoperative stay, and total length of stay were no different between time periods, Table 3. There was significant cost savings realized when both sites had an ACS service compared to when neither did, Table 3. When comparing influence on patient quality of life, all periods with an ACS service resulted in improved QALY over the fully non-ACS period. During period 2, the average per patient improved by 0.032 QALY (*p* < 0.001) vs period 1 and period 3 the average patient improved by 0.039 QALY (*p* = 0.013), Fig. 2. Regarding costs, implementation of ACS at site A during period 2 resulted in a non-significant average cost increase of $52 (*p* = 0.954). During period 3, when both sites had established ACS, there was a cost savings of $1162 (*p* = 0.05) per case compared to period 1. The primary outcome of ICER for period 2 compared to that for period 1 was $1626/QALY. The ICER for period 3 compared to that for period 1 was negative, and thus, the magnitude is not meaningful and therefore not reported (dominant strategy). Figure 2 displays a cost by QALY chart where the bottom right quadrant represents the ideal scenario of simultaneous cost saving with improved QALY outcomes. ACS at both sites (period 3) suggests both a significant cost saving and improved quality of life for patients.

There was an increase in Monday to Friday, daytime (7 a.m.–5 p.m.) operating room use, 46 to 62% when IC was performed, although this was not significant (*p* = 0. 32). There were more ultrasounds performed on patients undergoing an elective operation compared to IC; >25% of patients undergoing an elective operation required more than one ultrasound compared to <10% of patients undergoing an IC (*p* < 0. 001). ERCP rates were not statistically different between periods although less ERCPs were performed when both sites had ACS. Patients who did not undergo operative management were more likely to undergo ERCP compared to those undergoing operative management of GSP, 53% compared to 34% (*p* < 0.001).

## Discussion

The standard of care for uncomplicated GSP is IC, and our results confirm that the presence of ACS services can enable high rates of IC which are robust over time and replicable. Further, our results suggest that IC is exceptionally cost effective, resulting in both fiscal cost savings and improved patient quality of life outcomes. The most favorable ICER was the final period of study, where both sites had an ACS service, suggesting that ACS services deliver high quality and cost-effective care for GSP.

GSP management has shifted over the past decade from the previous preference for a “cooling off” period prior to outpatient cholecystectomy to the modern approach of performing early IC [23, 31–34]. The PONCHO trial, a randomized trial comparing IC to outpatient cholecystectomy confirms the safety and efficacy of definitive management for non-severe GSP on the index admission. Even with the short delay (25–30 days) for elective operation, 18% of patients in this study required re-admission for gallstone-related problems. Our re-admission rate for patients undergoing planned elective surgery was significantly higher, likely due to an even longer delay (194 days) to definitive management in this group. This was similar to the re-admission rate of ~30% seen in the group of patients who did not undergo surgical management. Our findings are consistent with other reports on mild non-severe gallstone pancreatitis [14, 15, 35]. Our previous work demonstrated that our institution had very low IC rates historically, and that the passage of time following the publication of mounting evidence and guidelines encouraging IC only marginally improved our IC rates [4]. It was clear, that at our institution, barriers to IC performance included a lack of operative resources accessible to emergency patients and competition with busy elective practices. Encountering intraoperative surgical difficulty with the “hot gallbladder” has been suggested as another potential additional barrier but has not been confirmed as a valid reason for delaying surgery. Numerous reports suggest cholecystectomy may be easier in the setting of GSP [36, 37]. Indeed, the PONCHO trial included a rating scale of intraoperative surgical difficulty and found no difference [4]. We also did not see an increase in operative time, length of stay, or rate of conversion associated with the increased rate of IC. Implementation of an ACS service with dedicated daytime emergency operative time allowed surgeons to suspend elective practice and facilitated IC achievement.

De Mestral et al. clearly demonstrate the variation in management of acute biliary disease. In this study, the authors found a fourfold difference in the likelihood of IC at various centers within the province of Ontario, Canada, even for young, healthy male patients with acute cholecystitis [25]. While our study represents some of the first work to demonstrate the increase of IC for uncomplicated GSP resulting from ACS adoption, others have demonstrated similar findings in acute cholecystitis. Pepingco et al. found an increase from 55 to 90% in the rate of IC for acute cholecystitis after the addition of an ACS service [5]. Our ACS service has dedicated daytime operating time at one site which we believe facilitates our improved IC rate. We obtained a rate of 76% without a subsequent increase in after-hours or weekend operations. Reports of operative timing for acute cholecystitis vary with some centers reporting an increase in after-hours/weekend operations [38], and others reporting a decrease [5, 9, 11]. Unfortunately, the role of a dedicated operating room is challenging to discern as the reporting on the design of the ACS services in the collective literature is generally poor.

Waiting for an elective operation, including cholecystectomy, significantly impacts quality of life from both potential physical and emotional aspects [39]. Further, quality of life can be impacted by the financial implications of missing work due to emergency room visits, outpatient clinic visits, and hospital re-admissions [40, 41]. For example, the number of patients reporting pain while waiting for surgery in the interval cholecystectomy group in the PONCHO trial was >50%. While other studies have demonstrated the cost savings associated with an ACS service for acute cholecystitis, none have done so for gallstone pancreatitis. We demonstrate significant cost savings of over $1000 per patient when both sites had an ACS service (period 3). This has significant health care resource utilization implications on a national scale given: (1) the number of cholecystectomies are performed on an annual basis and (2) gallstone-related disease is the leading cause of gastrointestinal admission to hospital [42]. Coupled with improved quality of life following cholecystectomy, the resulting negative incremental cost effectiveness ratio suggests both improvement of quality of life and a net cost benefit. Using the results of a Cochrane review on the management of GSP, Morris et al. demonstrated divergent results. In the analysis performed by Morris et al., early laparoscopic cholecystectomy (<72 h) demonstrated a cost reduction which was attributed to decreased length of stay [26]. In contrast, our cost savings results were largely driven by decreased re-admission rates, imaging utilization, and ER visits (while length of stay remained largely unchanged).

Our study is retrospective and is limited by the inherent biases associated with this study design, including identification of GSP patients. Further, we cannot be completely certain that the care of the study patients was contained to our institution and that they did not present elsewhere. Our electronic record does include most neighboring community hospitals and as such, we believe that the potential to miss re-presentations to nearby hospitals is very low but not impossible. Therefore, our results represent a minimum re-admission rate for GSP. In addition, we cannot adequately discern the reason for not undergoing IC and certainly mitigating factors such as patient preference may influence the timing of an operation. Finally, there are numerous other patient and societal factors that we cannot measure directly such as missed work and social welfare costs.

## Conclusions

A dedicated ACS service significantly improves rates of IC for GSP in a cost-effective manner that improves quality of life for patients. The increased rate of IC is associated with a similar magnitude in decreased re-admissions, ER visits, and ancillary testing. We believe that our results provide an important quality benchmark (rate of IC) and moreover, suggests that financial costs may no longer be (or at least less of) a barrier to the creation of ACS services than some may have previously thought. In fact, ACS adoption may allow surgical hospitals to achieve the “ultimate outcome”, generation of cost savings while providing better quality of care. General surgeons seeking to improve their care of emergency surgery patients via the adoption of an ACS service should consider our results when advocating and negotiating for resources with hospital administration and other stakeholders.

## Abbreviations

ACS: Acute care surgery
EGS: Emergency general surgery
ER: Emergency room
ERCP: Endoscopic retrograde cholangiopancreatography
GSP: Gallstone pancreatitis
IC: Index cholecystectomy
ICER: Incremental cost effectiveness ratio
LOS: Length of stay
OR: Operating room
QALY: Quality-adjusted life years
